# Gastrointestinal Symptoms and Elevated Levels of Anti-*Saccharomyces cerevisiae* Antibodies Are Associated with Higher Disease Activity in Colombian Patients with Spondyloarthritis

**DOI:** 10.1155/2017/4029584

**Published:** 2017-10-29

**Authors:** C. Romero-Sánchez, W. Bautista-Molano, V. Parra, J. De Avila, J. C. Rueda, J. M. Bello-Gualtero, J. Londoño, R. Valle-Oñate

**Affiliations:** ^1^Spondyloarthropathy Group, Rheumatology Department, Hospital Militar Central/Universidad de La Sabana, Chía, Colombia; ^2^Group of Applied Clinical Immunology Rheumatology and Immunology Department, Hospital Militar, Universidad Militar Nueva Granada, Bogotá, Colombia; ^3^UIBO Institute, Universidad El Bosque, Bogotá, Colombia; ^4^Faculty of Medicine, Universidad de La Sabana, Chía, Colombia; ^5^Spondyloarthropathy Group, Rheumatology Department, Hospital Militar Central, Faculty of Medicine, Universidad Militar Nueva Granada, Bogotá, Colombia

## Abstract

**Background:**

Spondyloarthritis (SpA) is a group of articular inflammatory rheumatic diseases that their gastrointestinal manifestations are around 10% of their extra-articular symptoms, supporting that the inflammatory response of the intestinal mucosa could be associated with the clinical status.

**Objectives:**

To investigate the association between gastrointestinal symptoms and autoantibodies and disease activity between SpA patients, healthy subjects (HS), and patients with inflammatory bowel disease (IBD).

**Methods:**

102 SpA patients, 29 IBD patients, and 117 HS were included. Autoantibodies as ASCA, ANCA, anti-tTG, anti-DGP, ANA, and IgA were measured. The patients were assessed to evaluate clinical and gastrointestinal symptoms. An association analysis was performed using Chi square test and a logistic regression.

**Results:**

Significant differences were found for ASCA levels in SpA (28.2%) compared to IBD (14.2%) and HS (6.0%) (*p* = 0.029), as well as for ANAS in SpA (49.5%) and IBD (37.9%) (*p* < 0.001) and abdominal pain (*p* = 0.012) between SpA (54.3%) and IBD (27.5%). Significant associations were found between BASDAI > 4 and gastrointestinal symptoms (*p* < 0.05) and IgA (*p* = 0.007). The association for abdominal bloating was maintained (OR: 3.93, CI-95%, 1.14–13.56; *p* = 0.030).

**Conclusions:**

Gastrointestinal symptoms, ASCA, ANAS, and IgA levels were associated with high disease activity in SpA compared with IBD and HS.

## 1. Introduction

Spondyloarthritis (SpA) comprises a group of rheumatic inflammatory chronic diseases that share clinical, radiographic, and immunogenic characteristics [1, 2]. This condition mainly affects males under 45 years of age and includes the following subtypes: ankylosing spondylitis (AS), reactive arthritis (ReA), psoriatic arthritis (PsA), inflammatory bowel disease associated with arthropathy, and undifferentiated spondyloarthritis (uSpA) [3]. The frequency of gastrointestinal manifestations in SpA ranges between 21 and 30% with a wide clinical spectrum. Approximately 5 to 10% of these manifestations are associated with inflammatory bowel disease (IBD). A substantial percentage of patients with unspecific gastrointestinal symptoms may have subclinical gut inflammation that may be confirmed only by endoscopy and histology [4, 5].

The association between gut and musculoskeletal system has been described previously. Wright and Watkinson in 1959 [6] and Wrigth and Watkinson in 1965 [7] described the clinical, radiologic, and serologic characteristics of a distinctive form of arthritis associated with IBD. They further proposed that SpA is not a disease without gastrointestinal involvement. In contrast, SpA may represent a spectrum of extraintestinal manifestations of inflammatory gastrointestinal diseases [8].

The immune dysfunction associated with the role of the microbiota of the gut mucosa is contributing factor to local morbidity [9]. An increase in bacterial growth, absorption of immune complexes, immune mediators (especially IL-6 and TNF-alpha), and gut permeability may be pathogenic determinants. The increase in gut permeability and abnormal levels of colonizing bacteria in active lesions of IBD may lead to absorption of proinflammatory bacterial components that stimulate a pathological immune response [10]. A similar pathogenesis has been discussed and proposed for SpA [11, 12].

It is interesting to consider the relationship between SpA, infection, and gut inflammation. Case reports have reported the clinical association of joint inflammation with the presence of* Shigella* sp.,* Yersinia* sp.,* Campylobacter* sp., or* Salmonella* sp. in the gut [11, 12]. Several studies have evidence of the involvement of gut mucosa and the development of SpA. However, there is scarce evidence about studies assessing gastrointestinal symptoms in patients with SpA without IBD [13]. Having this tool in daily practice would enable an earlier and more focused approach to modulate and influence joint manifestations. Therefore, the main objective of this study is to determine the frequency of gastrointestinal symptoms and the association with the presence of gastrointestinal autoantibodies in a group of patients with SpA and compare them with both patients with IBD and healthy subjects.

## 2. Materials and Methods

### 2.1. Study Population

A cross-sectional study was designed. The study population was divided into three groups: 103 patients with SpA according to the European Spondyloarthropathy Study Group (ESSG) [14], 29 patients with histological diagnosis of IBD according to the European Classification Consensus and the Montreal Classification for IBD [15, 16], and 117 healthy subjects. Those patients were enrolled from those attending regular rheumatology and proctology appointments at the Hospital from June 2012 to May 2014. Individuals with malignancies, autoinflammatory, or autoimmune diseases and antibiotic treatment in the past 3 months were excluded from the SpA group. Patients with irritable bowel syndrome with histopathology that ruled out IBD or other colitis with specific etiology (ischemic, infectious, or eosinophilic) were excluded from the IBD group. SpA patients with concomitant IBD were excluded. Unmatched 117 controls were included if they had no family history of SpA or IBD. The subjects were enrolled from either the same hospital or the patients' neighborhood. All patients and controls were between 18 and 65 years of age.

The patients were asked about the presence of clinical symptoms and the medical history was retrieved to find information of gastrointestinal symptoms: diarrhea (defined as more than three depositions per day), stools with mucous, hematochezia, number of stools per day, abdominal pain, abdominal bloating, food intolerances, and weight loss. The same rheumatologist evaluated all patients. These findings were compared to the Bath Ankylosing Spondylitis Functional Index (BASFI) and the Bath Ankylosing Spondylitis Disease Activity Index (BASDAI). The following laboratory tests were performed: ASCA (anti-*Saccharomyces cerevisiae* antibodies) IgG/IgA, 6 antigens associated with ANCA** (**anti-neutrophil cytoplasm antibodies), anti-tTG (anti-transglutaminase) IgG/IgA, anti-DGP (anti-deamidated gliadin peptide antibodies) IgG/IgA, ANAs (antinuclear antibodies), and total IgA.

### 2.2. Determination of ASCA (AESKULISA, Ref 3508 and 3507, Aesku.Diagnostics, Wendelsheim, DE)

Determination of anti IgG/IgA ASCAs antibodies was performed with an ELISA-based technique for quantitative detection in human serum using a positive reference value > 18 IU/ml. These determinations, when positive, should be conducted both independently (to determine which specific isotype is the one that reacts) and together with total IgA quantification because some patients with the disease have baseline deficiency of this immunoglobulin, indicating that interpretation must be based on IgG quantification data.

### 2.3. Determination of Anti-tTG and Anti-DGP (AESKULISA, Ref 3510, 3511, 3512, 3515, 3513, and 3514, Aesku.Diagnostics, Wendelsheim, DE)

A solid-phase immunoassay method was used to quantitatively detect IgG and IgA antibodies in serum against neoepitopes of tissue transglutaminase and antibodies directed against synthetic deaminated peptides derived from gliadin with positive reference values > 24 IU/ml.

### 2.4. Determination of ANCAS and ANAS by Indirect Immunofluorescence (IIF) AESKULISA, Ref 54.200, 54.201, and 55.100 (Aesku.Diagnostics, Wendelsheim, DE)

Neutrophil measurements were conducted on sheets fixed with ethanol and formalin by IIF assay for screening and semiquantitative determination of antibodies directed against the cytoplasm of polymorph nuclear neutrophils in human serum. The positive reference values for ANCAS were >1 : 20 and for ANAS were >1 : 80. ELISA confirmed all positive tests for ANCAS by IIF* (AESKULISA, Ref 3301, Aesku.Diagnostics, Wendelsheim, DE)* (elastase, lactoferrin, myeloperoxidase, proteinase 3, cathepsin G, lysozyme, and bactericidal permeability-increasing protein).

### 2.5. Activity Markers

Ultrasensitive C-reactive protein (CRP) was determined by chemiluminescence (Immulite 1000, Siemens®) REF LKCRP1; reference values 0–3 mg/dL. The sedimentation rate was automated in mm/hour by photometry test.

### 2.6. Total IgA Levels (Immage, Immunochemistry System, Beckman Coulter, Ref 446460 Brea, USA)

The levels were measured in serum using nephelometric kinetics with reference values. The biological reference interval for adults is 82 to 453 mg/dl.

### 2.7. Flow Cytometry Analysis to HLA-B27

Peripheral blood mononuclear cells were stained with monoclonal antibodies recognizing epitopes of HLA-B27 (anti-HLA-B27 fluorescein isothiocyanate (FITC)/CD3 phycoerythrin (PE) monoclonal antibody; Becton Dickinson, ref 340183, San Diego, USA). Lymphocytes were gated according to size and granularity and analyzed separately. Data were acquired using FACSCanto software, and the results were generated as graphics and format tabulations based on 15000 quantified events in the population of CD3. The positive results to HLA B-27 in the present study were confirmed by polymerase chain reaction with sequence-specific primer (PCR-SSP) methods (Biotest, Dreieich, DE). In the PCR-SSP methodology, primer pairs are designed to perfectly match only a single allele or group of alleles. Under strictly controlled PCR conditions, perfectly matched primer pairs result in the amplification of target sequences (i.e., a positive result), whereas mismatched primer pairs do not result in amplification (i.e., a negative result).

### 2.8. Statistical Analysis

A descriptive analysis and frequency distributions for clinical, laboratory, and demographic data were performed. Measures of central tendency and dispersion for clinical variables were obtained. A Chi-squared test was performed to determine associations. Comparisons between groups were performed using Kruskal-Wallis and Mann–Whitney *U*, given the previously confirmed nonparametric nature of the data. These inferential analyses were performed using IBM SPSS V20 software for Windows. A logistic regression was performed to adjust for possible confounding variables (age and sex) in the association between SpA and gastrointestinal outcomes. A stepwise function and a link test were used to validate the model, using STATA V12 for Windows.

The Hospital Militar Central Research and Ethics Committee approved this project (Reg. 2012-083). All patients approved and signed informed consent.

## 3. Results

### 3.1. Clinical and Laboratory Variables Associated with SpA

The median age of the participants was 40 years (IQR 28–52 years). Most of the participants were male (59.2%). The mean BASFI was 4.8 (SD ± 2.5). Sixty percent (60.6%) had BASFI > 4 and 36.54% > 6. The mean BASDAI was 5.2 (SD ± 2.2) with 67.7% above 4 and 41.1% above 6 and fatigue was present in 71.8%.

The median ESR was 16 mm^3^/h (IQR 8–28). Only 40.6% of the patients had a value above 20 mm^3^/h. The median CRP level was 0.33 mg/dL (IQR 0.1–0.9). Values above 3 mg/dL were present in 9.6% of the patients. Positive HLA-B27 was present in 39.6% of the patients. Regarding treatment in the SpA group, the patients were on antitumor necrosis factor (anti-TNF) medications, nonsteroid anti-inflammatory drugs (NSAID), sulfasalazine, or a combination of these medications.  Table 1 depicts the demographic characteristics of the studied population.

In total, 102 patients with SpA were included, of which 28.4% (*n* = 29) had AS, 56.9% (*n* = 58) had uSpA, and 14.7% (*n* = 15) ReA. Sixty-two percent of AS patients and sixty-three in uSpA and forty-six in ReA were males. The frequency of HLA B27 for the AS was 60.7%, for uSPA 32.7%, and for ReA 33.3% and other characteristics of the population by SpA subtypes are presented in Table 1.

### 3.2. Clinical Variables and Gastrointestinal Symptoms in SpA

The main gastrointestinal symptoms were abdominal bloating (54.9%), abdominal pain (54.9%), diarrhea (34.3%), bloody stool (14.7%), mucus in stool (20.6%), and weight loss (28.4%). Multiple food intolerance was found in 33.4% of the patients, followed by dairy intolerance (11.8%), vegetable protein (7.8%), and animal protein (4.9%) intolerance. Associations were established by Chi-squared test among SpA subsets. A relationship between mucus and ReA (*p* = 0.019) was observed. Abdominal bloating was associated with uSpA (*p* = 0.048), and a multiple food intolerance had a significative association with both uSpA and ReA (*p* = 0.044 and *p* = 0.020, resp.) (Table 2).

### 3.3. Autoantibodies and SpA

**Figure 1:**
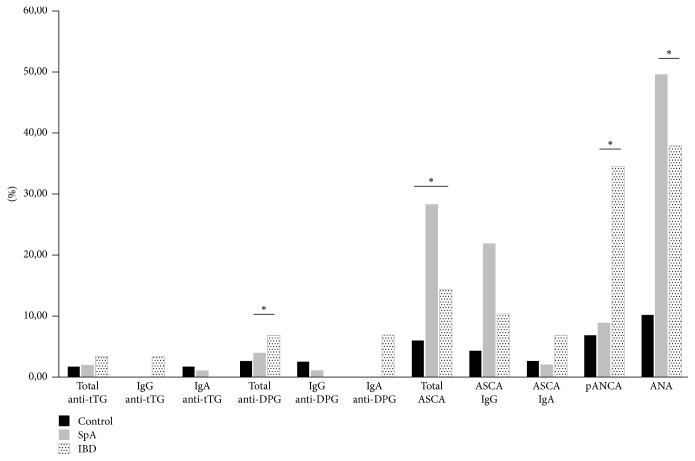
*Positive gastrointestinal autoantibody percentage in the studied population.* ^**∗**^ *Statistical associations by Chi square test*. The figure shows the frequency of positive autoantibodies according groups. A statistically significant difference was established between ASCA levels of the patients with SpA compared to the patients with IBD. Also, statistical significance was found between levels of anti-DPG IgA and IgG antibodies in the patients with IBD (44.89 IU/mL) compared to the SpA (16.91 IU/mL) patients. p-ANCA shows statistical associations with IBD, likewise, the ANAs with SpA.

Anti-tTG IgG/IgA antibodies were present in 1.9% and anti-DGP were present in 3.9% of the patients (median: 2.5 IU/mL, IQR 1.6–4.2; median: 4.5 IU/mL, IQR 3.2–7.5, resp.), and all were IgG isotype. Regarding ASCA antibodies, 21.8% were IgG isotype. Anti p-ANCA were positive in 8.8% of the patients, whereas ANAs were positive in 49.5% of the patients. The median value of IgA was 272 mg/dL (IQR 210–372). (Figure 1).

When autoantibodies were evaluated within the SpA groups, we found total ASCA in 12.1% in uSpA and 17.2% in AS. Of these groups, 47.6% were IgG, and 9.5% were IgA in uSpA, and 58.8% were IgG and 5.6% were IgA in AS. In the other groups of SpA, ASCAs were negative. Additionally, only 5.2% of the uSpA patients were positive for p-ANCA, whereas the AS patients had a higher frequency (13.8%). ANAS were almost equal in both SpA groups (uSpA: 48.3%, AS: 48.1%). Anti-tTG IgG and anti-DPG were negative in all SpA subtypes. Only one AS patient was positive for anti-tTG IgA (3.4%) and for anti-DPG IgA (3.4%).

### 3.4. Association between SpA Indexes and Gastrointestinal Symptoms

Associations were established by Chi-squared test between SpA indexes and gastrointestinal symptoms. Statistical significance was achieved for associations of high levels of BASFI (above 4) with abdominal bloating (69.6%; *p* = 0.028) and for associations of high levels of BASDAI (above 4) with diarrhea (41.4%; *p* = 0.027), abdominal pain (64.3%; *p* = 0.006), diarrhea (41.4%; *p* = 0.027), and abdominal bloating (66.7%; 0.001; OR 0.93 CI 95% 1.14–13.56; *p* = 0.030) (Table 3). Additionally, associations between higher levels of BASDAI (above 6), abdominal pain (54.5%; *p* = 0.003), and abdominal bloating (50.0%; *p* = 0.033) were statistically significant.

Another variable associated with levels of BASDAI > 4 with statistical significance was total serum level of IgA 244 mg/dL (194–337, *p* = 0.007), which retained significance for higher levels of BASDAI (above 6), *p* = 0.004. Levels of IgA were statistically significant with ASCAS IgG/IgA (26.4%; *p* = 0.014) and food intolerance (2.99%; *p* = 0.039).

### 3.5. Clinical Variables in IBD Patients

The group consisted of 29 patients: 24 with Ulcerative Colitis (UC) and 5 with Crohn's Disease (CD). The median age of the UC patients was 49.5 years (IQR 40.5–57). The mean weight was 72.8 kg (SD: ±13.6), and 50% of the patients were male. In the CD group, a higher proportion of male patients were found. Additionally, a lower mean age (36.2 years, SD: ±7.8) and weight (81 kg, SD: ±6.0) were found.

Regarding ESR, the patients with UC had a median of 23 mm^3^/h (IQR 8–40.2) and the patients with CD had a mean of 38.8 mm^3^/h (SD: ±24.2). In UC, the PCR was higher than in the CD patients (mean: 8.36 mg/dL; SD ± 27.9, and 1.3 mg/dL; SD ± 2.1, respectively).

Only 14.3% patients were positive for ASCA IgG/IgA (median 4.9 IU/mL, IQR 2.9–11.9), of which 6 were the IgG isotype, 3 were IgA, and 2 had both isotypes simultaneously. Regarding IBD, 15.8% of the patients were positive for ASCA in the UC group and 33.3% of the patients were positive in the CD group.

ANCA antibodies were found in 34.5% of the patients with UC and in 20% of the patients with CD. ANAS were positive in 37.9% patients, 12 with UC, and 2 with CD. Anti-tTG was positive in 3.4% and anti-DPG antibodies were positive in 6.9% in patients with IBD. For IgG/IgA autoantibodies interpretation, total IgA level was measured. Only 9% of the patients had total IgA above limit levels (median: 350 mg/dL, IQR 253–445). As for endoscopic severity, 38.7% of the patients with UC showed no involvement.

### 3.6. Clinical Variables in Healthy Subjects

The group consisted of 117 individuals with a median age of 50 years (IQR 28.5–68.7). The median of ESR and CRP was 6.5 mm^3^/h (IQR 4.0–12.0) and 0.16 mg/dL (IQR 0.10–0.28), respectively. ASCA antibodies had a median of 1.8 IU/mL (IQR 0.84−4.38), whereas total IgA had a median of 261.0 mg/dL (IQR 197.7–351.5). (Table 1).

### 3.7. Comparison between Studied Groups

A statistically significant difference was established for ASCA frequency between SpA patients (28.2%) compared to the patients with IBD (14.2%) and those with HC (6%) (*p* = 0.029). There was no statistical significance in ASCA distribution between uSpA (17.2%) and AS (12.1%). A significant increase was found for anti-DPG IgA/IgG antibodies in the patients with IBD compared to the SpA patients (44.89 IU/mL and 16.91 IU/mL, resp.) (*p* = 0.0001). A significant difference in the frequencies of p-ANCA among IBD (34.5%), SpA (8.8%), and HC (8.8%) (*p* < 0.001). A statistical difference was achieved (*p* = 0.0001) for ANAS between in SpA (49.5%) and IBD (37.9%) was observed. (Figure 1).

Comparing gastrointestinal symptoms, significant differences were observed for the presence of abdominal pain (*p* = 0.012) between SpA (54.3%) and IBD (27.5%), with a higher frequency in the SpA patients. In the analysis by SpA subtypes, 71% of the patients with ReA showed a predominant presence of mucus (*p* = 0.019), and a greater weight loss was detected in 34.5% of the patients with uSpA than in the other subtypes of SpA (*p* = 0.035).

## 4. Discussion

Our results have shown a higher frequency of gastrointestinal symptoms in patients with active SpA than in those with stable (non-active) disease. Additionally, differences in presentation according to SpA subtypes were observed. Patients with active SpA have higher levels of gastrointestinal autoantibodies than HS and even patients with IBD. Moreover, higher frequencies of autoantibodies associated with CD in SpA were observed, despite a higher frequency of UC in the Colombian population [17].

A study that included patients with AS have shown significantly higher levels of ASCA but not ANCAs compared with HS, suggesting a dysregulation in the gut mucosa as an important pathway in AS [18]. Similarly, in our study, higher levels of ASCA were observed in SpA patients than in HC patients; however, we did not find differences between ANCA levels in SpA in comparison to HC. In another study conducted in Belgium, levels of ASCA IgA/IgG and intestinal biopsies were performed in patients with CD, SpA (including patients with AS, uSpA, and ReA), and HS. They report that levels of ASCA IgA were significantly higher in SpA, specifically in AS without association with intestinal inflammation [19]. In contrast, patients with SpA showed higher levels of the IgG ASCA in comparison to the SpA patients in the study from Belgium.

Several studies have reported a frequency for ASCAs of 56% in CD and 14% in UC [20, 21]. Compared to those studies, our study found a lower frequency of 33.3% for patients with CD and a higher frequency in UC. Vergara et al. reported that the presence of ASCAs in UC occurs in patients with flares and colectomies, concluding that these patients have more disease severity. These biomarkers have proven to be useful in different forms of IBD, which combined with clinical, endoscopic, and radiologic data may be useful for diagnosis [21]. Elkadri et al. found, in a study of 391 patients with CD and 207 with UC, that positive values of ASCAs in CD and p-ANCAs in UC are associated with a more severe phenotype in CD and higher rates of colectomies in UC [22]. Our results differ from those data shown by Elkadri, in which Colombian population has a higher frequency of UC [23, 24] and higher levels of ASCAs. It can be associated with a different presentation of IBD in patients with SpA.

P-ANCAs are a heterogeneous group of autoantibodies of the IgG isotype generally associated more frequently with UC. García Herola et al. described a mean frequency of 40% for p-ANCA in UC and 17% in CD [25, 26]. Our results, in concordance with those reported in the literature, evidence a frequency for p-ANCA of 70% and 12.5% in UC and CD, respectively. None of the IBD and SpA patients recognized myeloperoxidase antigen, elastase, lactoferrin, proteinase 3, or cathepsin G, indicating that p-ANCA is a different antigen that is specifically recognized in vasculitis [26].

In previous studies, SpA have been associated with gut inflammation [27]. Histologic gut inflammation can be found in AS, uSpA, and ReA in approximately 30 to 60% of these patients [28]. Additionally, several studies have evidenced a close relationship of joint inflammation with the chronicity of gut inflammation. Cuvelier et al. found an association between chronic gut lesions and joint inflammation [29]. The prevalence of gut inflammation in AS patients has been found to be higher when peripheral arthritis is present [30]. The relationship has been further supported when Mielants et al. demonstrated a remission of joint inflammation in patients with a resolution of gut inflammation [31]. Additionally, chronic gut inflammation and mild complaints of diarrhea have been shown to be associated with a higher risk of evolution to AS in patients with IBD.

Since AS patients usually do not exhibit the typical clinical symptoms of inflammatory bowel disease such as frequent and severe diarrhea or bloody stools, these gastrointestinal complaints in AS patients have not been carefully evaluated. Most of these patients have been considered to be asymptomatic with regard to gastrointestinal manifestations. Data from 111 patients were available for analysis to investigate the relationship between diet and disease activity in a group of Swedish AS patients. Twenty-seven percent of these patients reported gastrointestinal pain and/or loose stools when consuming a particular foodstuff. The most common food stuff(s) to cause these symptoms were dairy products, vegetables/fruit, fatty foods, and food rich in flour [13]. Similar results were found in our study, multiple food intolerance followed by dairy intolerance, vegetable protein and animal protein intolerance.

It has been suggested that the heterogeneity of gastrointestinal complaints in SpA patients can be explained by genetic factors [32]. In addition, some data support that inflammation of the gastrointestinal tract may correlate with a more severe disease [33]. One study found that the 30% of patients reporting gastrointestinal pain had significantly higher disease activity and lower functional status according to the BASDAI and BASFI scores, respectively [13]. In the same way, statistical significance was observed regarding association between higher disease activity and lower function score with diarrhea, abdominal pain, and abdominal bloating.

A point of concern may be the lower frequency of HLA-B27 in Colombian general population and patients as compared to the frequency of HLA-B27 in patients in Europe and North America. Many studies have reported this finding not only in Colombia [32–35] but also in Latin America. This may be explained by differences with regard to ethnic and geographic variation. According to our results, the gastrointestinal manifestations are higher than previously reported in the literature [36]. This is mainly because this kind of complaints is not systematically investigated during clinical care in patients with SpA.

Our study had some limitations. First, the SpA subtype had different sample size. The total sample size overall is relatively small, which limits the external validity, generalizability, and the strength of association. Second, the cross-sectional design of the study may restrict the proposal of causal inferences. In this line, prospective studies with larger sample size are needed. Nevertheless, there are poor studies in the literature performed in patients with SpA and subsets that specifically focused on the evaluation of gastrointestinal symptoms.

In conclusion, we found a higher frequency of gastrointestinal symptoms and autoantibodies in patients with active SpA. An active search for gastrointestinal complaints and subclinical gut inflammation should be performed in patients with SpA in clinical practice. The evaluation of specific symptoms and gastrointestinal biomarkers might determine the patients that may benefit of endoscopic and histological evaluation.

## Figures and Tables

**Table 1:** Social and demographic description.

	SpA	AS+	uSpA+	ReA+	IBD	HS
	(*n* = 102)	(*n* = 29)	(*n* = 58)	(*n* = 15)	(*n* = 29)	(*n* = 117)
Age^†^	42.2 ± 15.1	48.4 ± 16.5	38.1 ± 13.3	37.3 ± 13.6	48.7 ± 13.3	41.2 ± 13.3
Gender (male)^§^	42 (40.7)	18 (62.1)	35 (60.3)	7 (46.7)	16 (50.0)	55 (47)
Weight^†^	69.9 ± 12.6	73.9 ± 11.6	69.5 ± 12.1	63 ± 13.7	72.8 ± 13.6	67.5 ± 12.9
HLA-B27^§^	40 (39.6)	17 (60.7)	18 (32.7)	5 (33.3)	N/A	5(4)^*∗*^
BASFI > 4^§^	60 (60.6)	18 (62.1)	38 (66.0)	8 (53.3)	N/A	N/A
BASDAI > 4^§^	67 (67.7)	19 (62.1)	41 (70.7)	11 (73.3)	N/A	N/A
ESR > 20 mm^3^/h^§^	39 (39.4)	17 (60.7)	20 (34.5)	7 (46.7)	26.7 (23.5)	9.9 (10.0)^*∗*^
CRP > 3 mg/Dl^§^	9 (9.6)	28 (96.6)	53 (91.4)	14 (93.3)	2.05 (3.5)	3.73 (4.8)^*∗*^
IgA (mg/dL)^‡^	272 (210–372)	251 (214–383)	272 (197–377)	270 (164–353)	329 (233–442)	274 (210–355)
Treatment						
NSAIDS^§^	24 (24.5)	5 (17.2)	17 (31.5)	1 (7.1)	None	N/A
Sulfasalazine^§^	8 (8.2)	1 (3.4)	6 (11.1)	2 (7.1)	1 (3.4)	N/A
Anti-TNF^§^	24 (24.5)	9 (31.0)	10 (18.5)	5 (35.7)	4 (13.7)	N/A
Combination with anti-TNF^§^	23 (23.5)	8 (27.6)	10 (18.5)	5 (35.7)	1 (3.4)	N/A
Sulfasalazine + NSAID^§^	19 (19.4)	6 (20.7)	11 (20.4)	2 (14.3)	None	N/A
Mesalazine^§^	None	None	None	None	21 (72.4)	N/A

^§^Results expressed as *n* (%). ^†^Results expressed as mean ± standard deviation. ^‡^Results expressed as median (interquartile range). BASFI: Bath Ankylosing Spondylitis Functional Index, BASDAI: Bath Ankylosing Spondylitis Disease Activity Index, Anti-TNF: antitumor necrosis factor, NSAID: nonsteroid anti-inflammatory drugs; N/A: not applied, ^*∗*^*p* < 0.05. +Diagnosis at the beginning of SpA diseases.

**Table 2:** Gastrointestinal characterization.

	SpA	AS	uSpA	ReA
	(*n* = 102)	(*n* = 29)	(*n* = 58)	(*n* = 15)
Diarrhea^§^	35 (34.3)	8 (27.6)	18 (31.0)	9 (60.0)
Blood^§^	15 (14.7)	4 (13.8)	9 (15.5)	2 (13.3)
Mucus^§^	21 (20.6)	6 (20.7)	8 (13.8)	7 (46.7)^*∗*^
Abdominal pain^§^	56 (54.9)	15 (51.7)	30 (51.7)	11 (73.3)
Abdominal bloating^§^	56 (54.9)	12 (41.4)	32 (56.1)^*∗*^	12 (80.0)
Weight loss^§^	29 (28.4)	3 (10.3)	20 (34.5)	6 (40.0)
Food intolerance				
Animal protein^§^	5 (4.9)	0 (0.0)	5 (14.3)	0 (0.0)
Vegetal protein^§^	8 (7.8)	1 (8.3)	7 (20.0)	0 (0.0)
Dairy^§^	12 (11.8)	6 (50.0)	4 (11.4)	2 (16.7)
Multiple^§^	34 (33.4)	5 (41.7)	19 (54.3)^*∗*^	10 (83.3)^*∗*^

^§^Results expressed as *n* (%). ^*∗*^Statistical association by Chi-square test (*p* < 0.05).

**Table 3:** Association between BASDAI^*∗*^ and gastrointestinal symptoms in SpA population.

	Inactive ≤ 4	Active > 4	*p* value
	(*n* = 33)	(*n* = 69)	
Abdominal pain^§^	11 (33.3)	45 (64.3)	0.006
Abdominal bloating^§^	10 (30.3)	46 (66.7)	0.001
Diarrhea^§^	6 (18.2)	29 (41.4)	0.027
Food intolerance^§^	13 (39.4)	47 (73.5)	0.025
IgA (g/dL)^‡^	339.5 (246.5–435.0)	244 (194.0–337.0)	0.007

^§^Results expressed as *n* (%). ^‡^Results expressed as median (interquartile range). ^*∗*^BASDAI: Bath Ankylosing Spondylitis Disease Activity Index.
